# A Machine Learning Model for Risk Stratification of Postdiagnosis Diabetic Ketoacidosis Hospitalization in Pediatric Type 1 Diabetes: Retrospective Study

**DOI:** 10.2196/53338

**Published:** 2024-08-07

**Authors:** Devika Subramanian, Rona Sonabend, Ila Singh

**Affiliations:** 1 Department of Computer Science Rice University Houston, TX United States; 2 Department of Pediatrics Baylor College of Medicine Houston, TX United States; 3 Division of Endocrinology Department of Pediatrics Texas Children’s Hospital Houston, TX United States; 4 Department of Pathology and Immunology Baylor College of Medicine Houston, TX United States; 5 Divisions of Laboratory Medicine and Pathology Informatics Department of Pathology Texas Children’s Hospital Houston, TX United States

**Keywords:** pediatric type 1 diabetes, postdiagnosis diabetic ketoacidosis, risk prediction and stratification, XGBoost, Shapley values, ketoacidosis, risks, predict, prediction, predictive, gradient-boosted ensemble model, diabetes, pediatrics, children, machine learning

## Abstract

**Background:**

Diabetic ketoacidosis (DKA) is the leading cause of morbidity and mortality in pediatric type 1 diabetes (T1D), occurring in approximately 20% of patients, with an economic cost of $5.1 billion/year in the United States. Despite multiple risk factors for postdiagnosis DKA, there is still a need for explainable, clinic-ready models that accurately predict DKA hospitalization in established patients with pediatric T1D.

**Objective:**

We aimed to develop an interpretable machine learning model to predict the risk of postdiagnosis DKA hospitalization in children with T1D using routinely collected time-series of electronic health record (EHR) data.

**Methods:**

We conducted a retrospective case-control study using EHR data from 1787 patients from among 3794 patients with T1D treated at a large tertiary care US pediatric health system from January 2010 to June 2018. We trained a state-of-the-art; explainable, gradient-boosted ensemble (XGBoost) of decision trees with 44 regularly collected EHR features to predict postdiagnosis DKA. We measured the model’s predictive performance using the area under the receiver operating characteristic curve–weighted *F*_1_-score, weighted precision, and recall, in a 5-fold cross-validation setting. We analyzed Shapley values to interpret the learned model and gain insight into its predictions.

**Results:**

Our model distinguished the cohort that develops DKA postdiagnosis from the one that does not (P<.001). It predicted postdiagnosis DKA risk with an area under the receiver operating characteristic curve of 0.80 (SD 0.04), a weighted *F*_1_-score of 0.78 (SD 0.04), and a weighted precision and recall of 0.83 (SD 0.03) and 0.76 (SD 0.05) respectively, using a relatively short history of data from routine clinic follow-ups post diagnosis. On analyzing Shapley values of the model output, we identified key risk factors predicting postdiagnosis DKA both at the cohort and individual levels. We observed sharp changes in postdiagnosis DKA risk with respect to 2 key features (diabetes age and glycated hemoglobin at 12 months), yielding time intervals and glycated hemoglobin cutoffs for potential intervention. By clustering model-generated Shapley values, we automatically stratified the cohort into 3 groups with 5%, 20%, and 48% risk of postdiagnosis DKA.

**Conclusions:**

We have built an explainable, predictive, machine learning model with potential for integration into clinical workflow. The model risk-stratifies patients with pediatric T1D and identifies patients with the highest postdiagnosis DKA risk using limited follow-up data starting from the time of diagnosis. The model identifies key time points and risk factors to direct clinical interventions at both the individual and cohort levels. Further research with data from multiple hospital systems can help us assess how well our model generalizes to other populations. The clinical importance of our work is that the model can predict patients most at risk for postdiagnosis DKA and identify preventive interventions based on mitigation of individualized risk factors.

## Introduction

### Background

Diabetic ketoacidosis (DKA) is the leading cause of morbidity and mortality among patients with pediatric type 1 diabetes (T1D), accounting for nearly 50% of all deaths in this population [1,2]. DKA occurs in 20% of patients with T1D, with an average cost of US $26,566 per DKA admission and a total economic cost of US $5.1 billion/year in the United States [3-7]. Hospitalizations for DKA in the United States have increased by 6.3% each year from 2009 to 2014 despite many attempts at prevention [8]. The incidence of DKA hospitalizations postdiagnosis has been estimated to be about 8 to 16 per 100 person-years in the pediatric population, with variations in both patient populations and in hospital or care systems [9]. DKA has a significant impact on growth and development in children, potentially leading to neurocognitive impairment, cerebral edema, coma, or even death [1,2,10].

Most prior studies pertaining to DKA hospitalization risk in pediatric patients are associational in nature, focusing on assessing DKA prevalence, predicting the risk of DKA at onset, and relating DKA at onset to its impact on glycemic control. These studies [11-18], conducted with a limited number of electronic health record (EHR)–derived features, using classical statistical methods, have identified the most common factors associated with DKA in patients with pediatric T1D. They include (1) insulin omission, especially in the context of chronic hyperglycemia (high glycated hemoglobin [HbA_1c_]) [5], (2) females of age greater than 10 years, (3) racial minority youths (Hispanic and African American) [19-21], (4) nonprivate health insurance (a proxy for socioeconomic disadvantage) [22,23], (5) underlying mental health comorbidities, and (6) prior-DKA [19,23-25].

Despite knowledge of DKA risk factors, there are few predictive tools ready for clinical integration that can accurately stratify DKA risk for established patients. This is partly because the relationship between known risk factors and postdiagnosis DKA is complex [4,20,25] and highly nonlinear, whereas tools for elucidating them have been generally limited to simple statistical models, such as logistic regression. Over the last 2 decades, nonlinear predictive techniques ranging from deep neural networks [26] to ensemble methods such as bagging and boosting [27], have been devised in the field of supervised machine learning. These methods derive their power from the ability to infer complex prediction functions directly from raw data. They have allowed for great progress in some diagnostic areas: diabetic retinopathy [28], machine translation of clinical notes [29], object recognition in radiologic or pathologic images [30], as well as in DKA prediction in both patients with pediatric and adult T1D [31,32], but pose challenges in terms of interpretability.

### Objective

We develop an explainable, machine-learning model to predict pediatric patients with T1D who are at risk of DKA hospitalization postdiagnosis using a time-series of routinely collected, EHR data. We evaluate the predictive performance of our gradient-boosted decision tree model (XGBoost) on one of the largest cohorts of pediatric patients with T1D. Further, we use Shapely value analysis of our model outputs to (1) derive key predictive factors for postdiagnosis DKA, both at the cohort and at the individual levels, (2) reveal the progression of postdiagnosis DKA risk over time, and (3) automatically perform cohort-level risk stratification by agglomerative clustering of Shapley values.

## Methods

### Study Design

This study accessed deidentified EHR data from 6288 pediatric patients with diabetes, 3794 of them with a confirmed T1D diagnosis, between January 1, 2010, and June 30, 2018, treated at Texas Children’s Hospital (TCH). TCH is one of the largest tertiary-care pediatric health systems in the United States, and likely has some of the largest sets of pediatric patients with diabetes.

To limit unintended biases and erroneous predictions caused by missing data, we defined stringent inclusion criteria to select the training cohort for model building. We selected patients who were (1) initially diagnosed at, and subsequently followed up within the TCH system with an onset date on or after January 1, 2010, (2) whose age at diagnosis was between 0 and 21 years, (3) who had at least 1 positive antibody titer (glutamic acid decarboxylase 65-kilodalton isoform [GAD65], islet cell autoantigen 512 [ICA512], and insulin AB) at diagnosis, and (4) with a clinical diagnosis of T1D by an endocrinologist. These criteria excluded 1723 patients from the first criterion, 45 from the second, and 239 from the third; with a remainder of 1787 patients included in the final analysis. Of these 1787 patients, 324 experienced at least 1 postdiagnosis DKA hospitalization. The small number of overall and positive cases is a consequence of T1D itself being a rare pediatric disease and postdiagnosis DKA being a rare complication of this disease.

### Feature Generation and Selection

#### Feature Construction

For each patient in the cohort, we extracted more than 100 features available in the EHR, from the time of diagnosis to 3-month clinic follow-ups for up to 2 years after onset. The data included demographic information, clinical data, laboratory values, treatment modality (insulin delivery), hospitalization records, and ambulatory care components. Demographic features included age at diagnosis (onset age), sex, race, ethnicity, and socioeconomic status proxies such as type of insurance, and zip code of residence. Clinical features included vital signs, BMI, and laboratory values including titers at the time of diagnosis. We included both raw diabetes titer values, as well as discretized Boolean (0 or 1) titer values (1 if GAD65 [33] titer >5 IU/mL, 1 if ICA512 [33] titer >5.4 IU/mL, 1 if insulin AB [33] titer >0.4 U/mL). We also included HbA_1c_ values at diagnosis and at 3-month clinic follow-ups for up to 24 months. Hospitalization features included length of stay, laboratory-test time-series during the stay, as well as therapeutic interventions. Ambulatory care features included the use of auxiliary services (educators, nutrition services, psychology, and social workers), as well as no-shows and cancellations. We also included diabetes age (years after T1D onset), whether there was DKA at onset, and C-peptide value at the time of diagnosis since they were clinically relevant features.

#### Feature Selection

We omitted features that were missing values for more than 50% of the cohort. We dropped ambulatory care component features and most laboratory test features (except for HbA_1c_) on this basis. We also omitted features highly correlated with HbA_1c_ values (such as the BMI time series) because they did not add to the predictive power of the model. In addition, we dropped all features perfectly correlated with the outcome variable—these included all hospitalization-derived features including laboratory tests conducted during DKA hospitalization and therapeutic interventions during hospitalization. This left us with 44 features described in detail later. We did not use additional feature selection methods, relying instead on XGBoost to select relevant features in the construction of the final decision ensemble.

#### Missing Value Imputation

We used a simple piecewise linear interpolation technique to fill in missing values in HbA_1c_ records. HbA_1c_ imputation was done only between 2 known values—for example, if the 3-month and 9-month HbA_1c_ values for a patient were known, then the 6-month value was imputed as the average. We did not perform any other imputation. The XGBoost learning algorithm handles missing values by default, obviating the need for more imputation.

#### Final Features

The 44 features finally used for each patient in our cohort included 15 demographic features: sex (male or female), insurance (private or Medicaid or self-pay), race (White, African American, Asian, and Other), ethnicity (Hispanic, non-Hispanic, and Other), first 3 digits of zip code, 7 diabetes titers (raw values and discretized values for GAD65, ICA512, insulin AB, and the total number of positive antibody titers), 17 HbA_1c_ features including 9 values of HbA_1c_ (at diagnosis, and at 3-month follow-ups from 3 to 24 months), as well as 8 delta measures (differences between HbA_1c_ measurements at successive follow-ups), and 5 other features: diabetes age (years since T1D diagnosis), onset age, DKA at onset (yes or no), C-peptide titer at diagnosis, and discretized C-peptide (>1 U/mL).

### Outcome Definition

We used hospitalization with DKA after diagnosis of T1D to define the outcome variable. We split the cohort into 2 classes: those who experienced at least 1 DKA episode after diagnosis (324/1787, 18%) and those who did not.

### Model Selection and Training Protocol

We trained a multivariate gradient boosting decision tree ensemble on the data, using the Python XGBoost open-source library [34]. As illustrated in Figure S1 in Multimedia Appendix 1, we used a 5-fold stratified cross-validation approach. We divided the data set into 5 equal-sized folds, with the ratio of patients with postdiagnosis DKA and non-DKA being equal in all groups. We used 4 of the folds for training a gradient-boosted ensemble and used the held-out fold for testing the ensemble. We repeated the process 5 times, each time with a different held-out fold, yielding 5 sets of performance measures. We used 4 standard metrics to quantify the performance of the postdiagnosis DKA classifier: area under the receiver operating characteristic curve (AUC), weighted *F*_1_-score, weighted precision, and weighted recall. We reported the mean and SD of these 4 scores across the 5 folds, to characterize the predictive performance of the ensemble model.

Key hyper-parameters for XGBoost (number of trees and tree depth) were selected using the standard hyperparameter tuning process described in section 5.3 of *Deep Learning* by Goodfellow et al [35]. We held out 10% of the training data in each cross-validation fold as a validation set—this data does not participate in model construction.

One of the challenges in model training is handling class imbalance (324 positive examples in a set of 1787 patients, in our case). This is an inherent consequence of postdiagnosis DKA being relatively uncommon. XGBoost handles imbalanced data sets by using the parameter scale_pos_weight, to reflect the degree of imbalance. This parameter weights the components of the cross-entropy loss function used by the training algorithm, assigning a higher weight to the minority class examples, in effect, simulating the process of up-sampling the minority class [36].

### Explaining Classifier Performance: Bee-Swarm and Main-Effects Plots

We used the Shapley value [37] framework to assign predictive importance to each feature. The Shapley value, or SHAP (Shapley additive explanations) value, of a feature for a patient, is a quantification of the contribution made by that feature to the DKA or no-DKA prediction made for that patient. The unit of measurement for SHAP values is the change in logarithmic odds of postdiagnosis DKA with and without the feature. Positive SHAP values mean a positive impact on prediction, that is, they lead the model to predict postdiagnosis DKA. Negative SHAP values mean a negative impact on prediction, that is, they lead the model to predict no DKA after diagnosis. Unlike regular feature importance plots, SHAP values show the directionality of the impact of the feature value on the outcome. We use plots of averaged SHAP values over the whole cohort for each feature, called a bee-swarm plot, to rank key factors that determine the risk of postdiagnosis DKA at the level of the entire cohort.

Main-effects plots show variation in the log-odds of postdiagnosis DKA as functions of a single predictor, all else being equal. A sharp change in log-odds in the main-effects plot of a feature reveals important thresholds at which postdiagnosis DKA risk increases or decreases. For example, these plots help answer questions such as: does the risk of postdiagnosis DKA increase linearly with diabetes age, or is there an age interval where the risk rises significantly? For an individual patient, SHAP values allow for the selection of features relevant to the prediction outcome and explain the outcome as an additive combination of SHAP values. We produce main-effects plots for key cohort-level risk factors identified in the bee-swarm plot.

### Cohort Risk Stratification by Clustering Shapley Values

Cohort risk stratification is a byproduct of the Shapley value analysis. We clustered the Shapley value matrix constructed from the output of the XGBoost model using a hierarchical agglomerative clustering algorithm, based on Ward linkage [38]. The algorithm groups patients according to similarities in their Shapley value vectors, producing a dendrogram. Any horizontal cut of the dendrogram induces a clustering of the original data. We select a cut level to maximize the dissimilarity between clusters.

### Personalized Risk Assessment

In addition to cohort-level predictions, the model is equally useful for generating interpretable predictions for individual patients. The model produces an additive risk score, which is the sum of the Shapley values for each predictive feature for that patient. When the sum is positive, it indicates higher than baseline risk for that patient; if it is negative, then the patient is at lower risk relative to the whole cohort.

### Ethical Considerations

Data were gathered under institutional review board (number H-42624), which was approved by the institutional review board of Baylor College of Medicine. The institutional review board covers secondary analysis utilizing this data without additional consent. Data were deidentified prior to analysis.

## Results

### Descriptive Analysis of Data

We summarize the value distributions of key predictors in Table 1. Surprisingly, DKA at the onset which has been shown to be associated historically with worsening glycemic control over time [13], does not have a strong correlation with postdiagnosis DKA. The median diabetes age at the first DKA after diagnosis is 2.43 (IQR 1.26-4.09) years. It validates the selection of time-series of HbA_1c_ measurements from baseline to the first 24 months after diagnosis, as the basis for postdiagnosis DKA prediction.

**Table 1:** Value distributions of key demographics and laboratory test values for the entire cohort of 1787 patients with type 1 diabetes treated at Texas Children’s Hospital between January 1, 2010, and June 30, 2018.

Feature		Variables
Sex (female), n (%)		891 (49.86)
**Race, n (%)**		
	African American	299 (16.73)
	Asian	65 (3.64)
	White	1364 (76.33)
	Other	59 (3.30)
**Ethnicity, n (%)**		
	Non-Hispanic	1301 (72.80)
	Hispanic	442 (24.73)
	Other	44 (2.46)
**Insurance, n (%)**		
	Private	1146 (64.13)
	Medicaid	641 (35.87)
**Antibody titer, median (IQR)**		
	Glutamic acid decarboxylase 65-kilodalton isoform	13.0 (3.85-30.00)
	Islet cell autoantigen 512	6.80 (1.2-20.8)
	Insulin antibody	0.4 (0.4-3.50)
**HbA_1c_^a^, median (IQR)**		
	HbA_1c_ baseline at diagnosis (n=1787)	11.1 (9.5-12.90)
	HbA_1c_ at 3 months (n=1768)	7.51 (6.76-8.48)
	HbA_1c_ at 6 months (n=1712)	7.34 (6.52-8.37)
	HbA_1c_ at 9 months (n=1651)	7.71 (6.87-8.7)
	HbA_1c_ at 12 months (n=1553)	7.85 (7.08-8.84)
	HbA_1c_ at 15 months (n=1471)	7.95 (7.20-8.91)
	HbA_1c_ at 18 months (n=1401)	8.07 (7.30-9.07)
	HbA_1c_ at 21 months (n=1320)	8.13 (7.31-9.10)
	HbA_1c_ at 24 months (n=1239)	8.15 (7.33-9.20)
C-peptide, median (IQR)		0.43 (0.26-0.725)
Duration of T1D^b^ (in years; diabetes age), median (IQR)		4.10 (1.80-6.47)
Age at T1D diagnosis (in years; onset age), median (IQR)		10.40 (6.73-13.43)
DKA^c^ at onset, n (%)		623 (34.86)
Diabetes age at first DKA postdiagnosis (years postdiagnosis), median (IQR)		2.43 (1.26-4.09)
Patients with at least 1 postdiagnosis DKA, n (%)		324 (18.13)

^a^HbA_1c_: glycated hemoglobin.

^b^T1D: type 1 diabetes.

^c^DKA: diabetic ketoacidosis.

### Model Evaluation by Cross-Validation With AUC, Precision, and Recall

**Figure 1:**
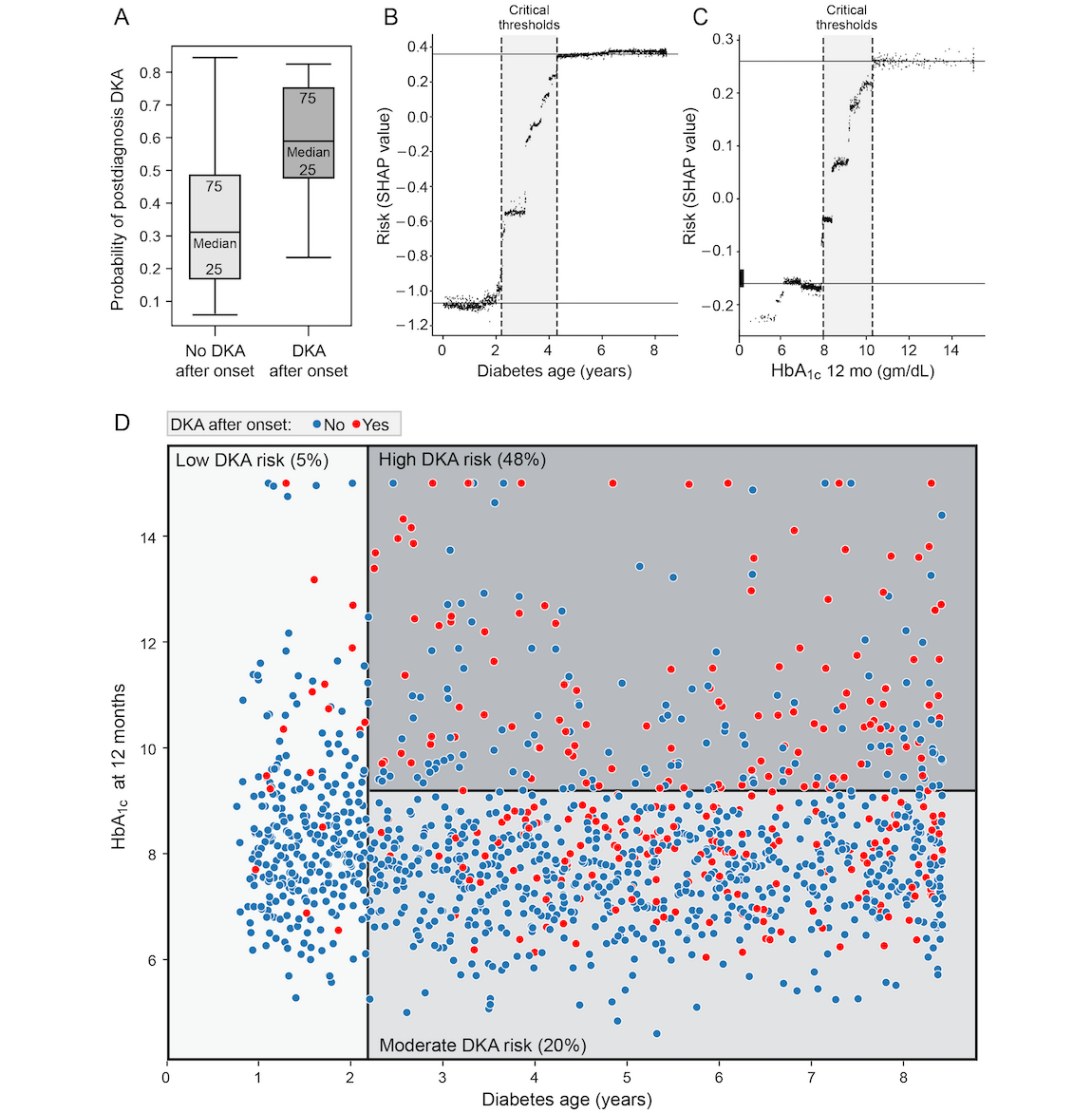
(A) Probability of postdiagnosis DKA at the cohort level, (B,C) risk by individual key features of diabetes age and HbA_1c_ at 12 months, and (D) risk stratification into group. The cohort that will develop DKA can be distinguished from the one that will not, with a (A) *P* value <.001. The main-effects plots show critical thresholds where risk sharply rises (gray regions), (B) for diabetes age between 2.2 and 4.3 years, and (C) for HbA_1c_ at 12 months between 8 and 10.3. (D) The scatterplot uses a diabetes age cutoff of 2.2 years and HbA_1c_ at 12 months of 9.2 to stratify the population into 3 risk groups for postdiagnosis DKA. A total of 30% of the population has 5% or low risk for DKA, shown in light gray; 50% of the population is at 20% or moderate risk (in medium gray), and 20% is at 48% or high risk of postdiagnosis DKA. DKA: diabetic ketoacidosis; HbA_1c_: glycated hemoglobin.

The box plot in Figure 1A shows the clear separation in probability between the no DKA and DKA postdiagnosis cohort (P value <.001). The model is able to distinguish between the postdiagnosis DKA cohort and the non-DKA cohort at a statistically significant level. The cross-validated model with all 9 HbA_1c_ measurements from baseline to 24 months has an AUC of 0.80 (SD 0.04) and a weighted *F*_1_-score of 0.78 (SD 0.04). The weighted precision and recall of the model are 0.83 (SD 0.02) and 0.76 (SD 0.07) respectively. Cross-validation allows robust estimation of the predictive performance of the model on new patients. Table S1 in Multimedia Appendix 1 shows the incremental effect of the addition of HbA_1c_ values from 3 to 24 months on these performance measures. Model performance stops improving after the addition of HbA_1c_ at 18 months after onset.

### Key Predictors of Postdiagnosis DKA

**Figure 2:**
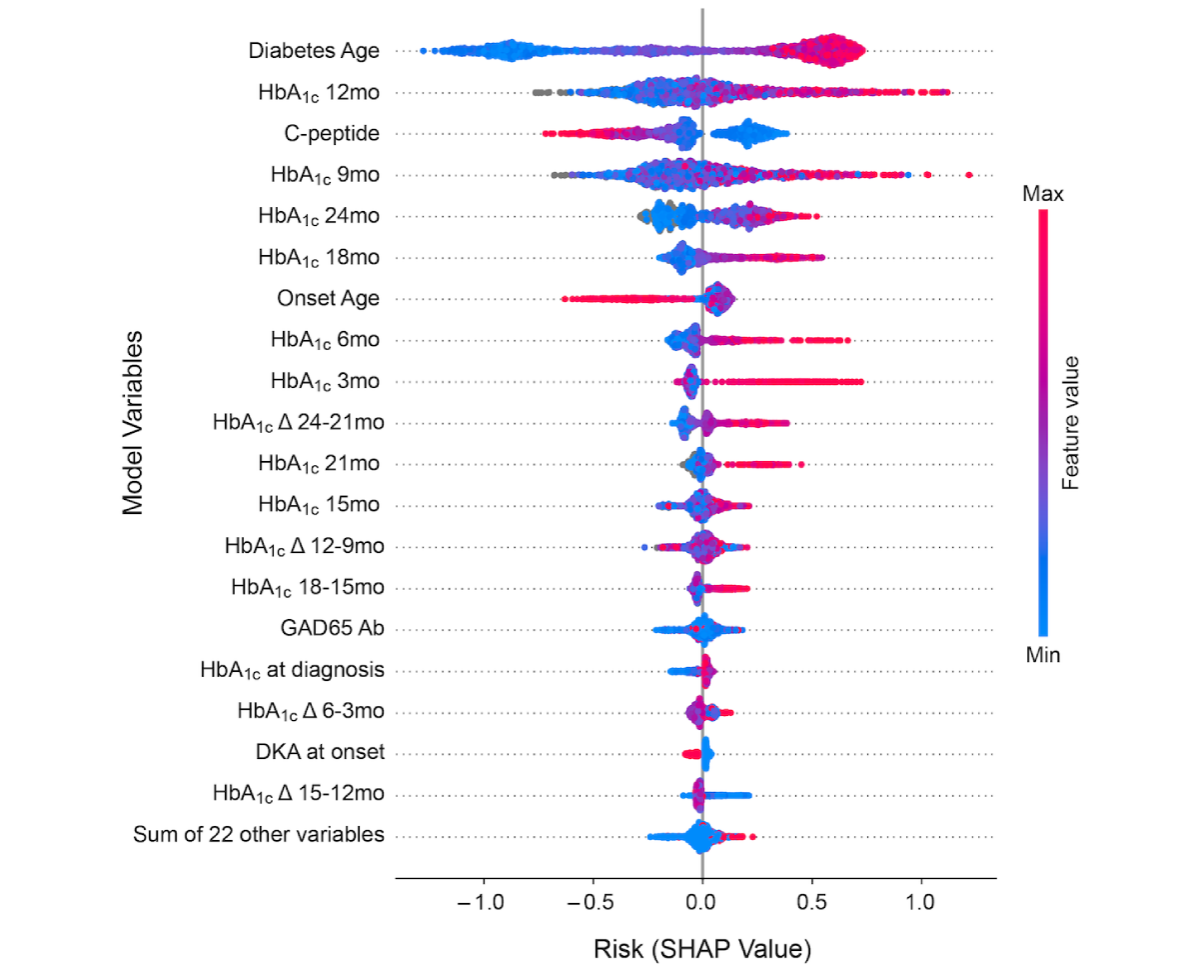
Model features displayed in order of decreasing importance for postdiagnosis DKA risk prediction. Each feature in this bee-swarm plot is shown with its full range of values—from a minimum in blue, to a maximum in red; see the color scale on right for values in between. The x-axis defines the log-odds of postdiagnosis DKA risk (SHAP value). Each colored dot in a horizontal line represents the value of the corresponding feature for a patient in the training cohort. The most important predictors are diabetes age, HbA_1c_ at 12 months, and C-peptide titer at diagnosis. DKA: diabetic ketoacidosis; GAD65: glutamic acid decarboxylase 65-kilodalton isoform; HbA_1c_: glycated hemoglobin; SHAP: Shapley additive explanations.

Figure 2 shows a bee-swarm plot summarizing the entire distribution of SHAP values for each predictor. The x-axis of the plot shows the impact on model output (log-odds of postdiagnosis DKA risk) of each of the predictors sorted along the y-axis by decreasing importance. The most important feature that predicts postdiagnosis DKA is diabetes age (top row). Every point in the top row denotes a patient’s diabetes age and the impact of the value of diabetes age on their log-odds of postdiagnosis DKA risk. Patients with low diabetes age (newly diagnosed, and colored blue) have negative SHAP values and thus lower postdiagnosis DKA risk, while patients with higher diabetes age (colored red) have positive SHAP values and higher postdiagnosis DKA risk. As diabetes age increases, the log-odds of DKA risk goes from –1.25 to +0.7. While the plot shows the overall trend of increasing diabetes age contributing to increased risk of postdiagnosis DKA it does not elucidate the exact nature of that trend. HbA_1c_ value at 12 months is the second most important feature, and the plot shows a trend of increasing postdiagnosis DKA risk with an increase in HbA_1c_ values. For the third most important feature, C-peptide at diagnosis, the plot shows that higher values at diagnosis are associated with lower postdiagnosis DKA risk. Higher HbA_1c_ levels at 9, 18, and 24 months are all associated with higher postdiagnosis risk. Higher onset age, ranked seventh in the ordering, is associated with lower postdiagnosis DKA risk. Not only has the model identified and ranked key predictors, but it also provides a quantitative measure of the impact of each of these features on the probability of postdiagnosis DKA risk.

Figures 1B and 1C show the main effects of 2 of the top predictors in the model: diabetes age and HbA_1c_ at 12 months. The log-odds of postdiagnosis DKA risk do not vary linearly with diabetes age. Rather, there is a threshold effect, with a rapid increase in log-odds of risk from –0.95 to +0.35 units between diabetes ages of 2.2 and 4.3 years. HbA_1c_ levels at 12 months reveal a similar threshold effect: levels below 8 are at relatively low risk of postdiagnosis DKA, with the risk rising steeply from –0.15 to +0.26 units for patients with HbA_1c_ levels between 8 and 10.3. Beyond the value of 10.3, the risk contribution of the 12-month-HbA_1c_ level plateaus at a log-odds of 0.26. Taken together, as shown in the shaded regions of the plots, the model predicts that at the cohort level, diabetes age between 2.2 and 4.3 years, and HbA_1c_ levels at 12 months between 8 and 10.3 offer the best intervention points to influence postdiagnosis DKA risk.

### Cohort Risk Stratification by Clustering Shapley Values

The agglomerative clustering analysis reveals 3 well-separated groups characterized by postdiagnosis DKA rates of 5%, 20%, and 48% respectively. Note that the clustering algorithm does not have access to the features or the labels (DKA or non-DKA) for each patient, but only the Shapley value of each feature for that patient. By using a decision tree algorithm to predict cluster membership, we identified the primary criteria to be a diabetes age cutoff of 2.2 years and an HbA_1c_ at a 12-month cutoff of 9.2. Figure 1D visually displays these clusters across the dimensions of diabetes age and HbA_1c_ at 12 months. Cluster 1, which is the low-risk group (5% probability of postdiagnosis DKA) consists of patients whose diabetes age is less than 2.2 years. Cluster 2, the medium risk group (20% probability of postdiagnosis DKA) is composed of patients with diabetes age of 2.2 years and older and HbA_1c_ at 12 months <9.2. Cluster 3, the high-risk group (48% probability of postdiagnosis DKA) consists of patients with diabetes age of 2.2 years and older and HbA_1c_ at 12 months>9.2. Patients at low risk constitute 30% of the population, those at medium risk constitute 50% of the population, and the high-risk group constitutes 20% of the population.

### Personalized Risk Assessment

**Figure 3:**
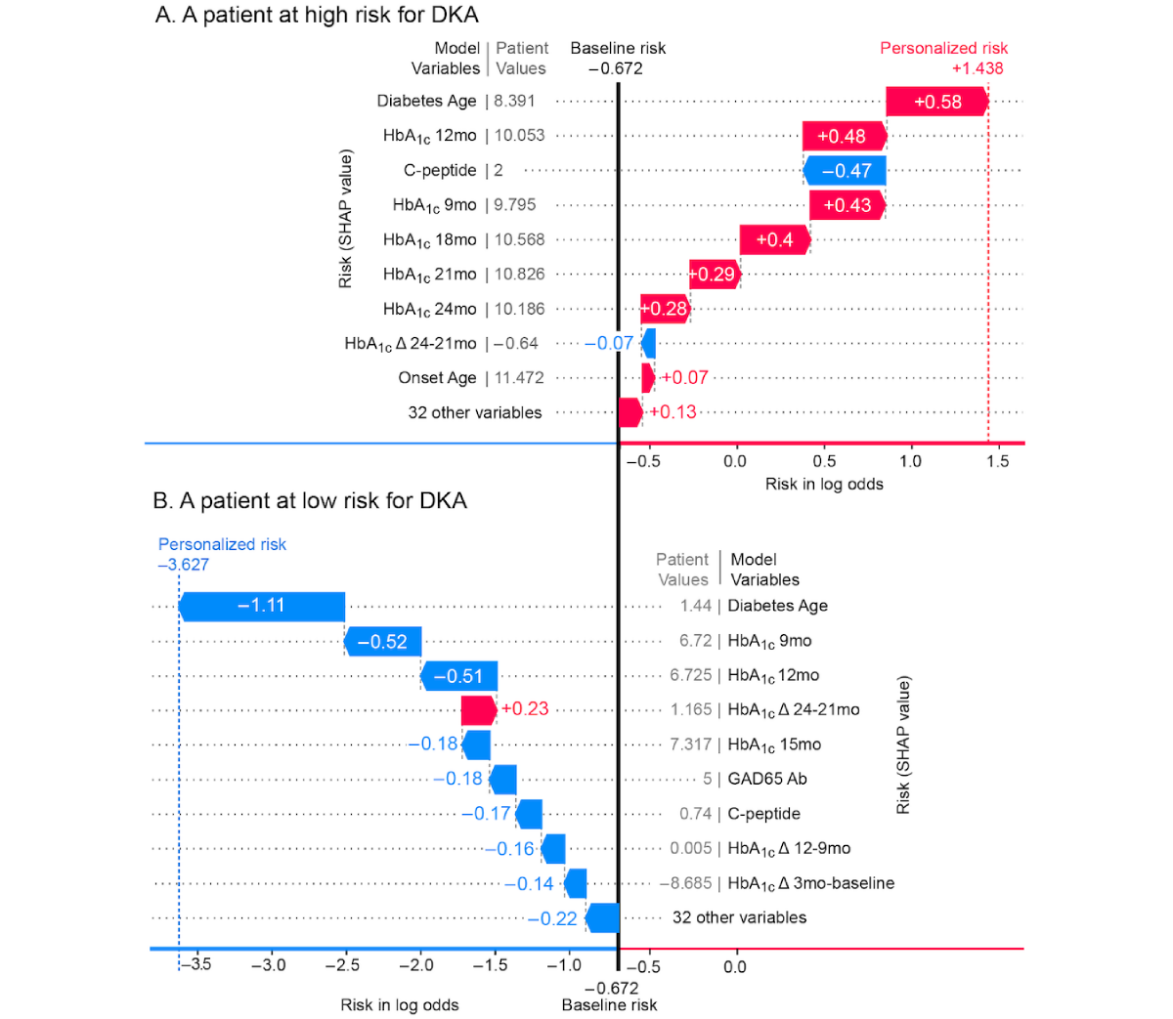
Risk models for 2 individual patients (A) at high risk and (B) at low risk of postdiagnosis DKA shown as waterfall plots. The baseline risk of –0.672 marked with a black vertical line represents the overall risk of postdiagnosis DKA at the cohort level. Factors in red are associated with higher DKA risk, and those in blue are associated with lower risk. The personalized risk score represents an individual’s unique risk profile. DKA: diabetic ketoacidosis; GAD65: glutamic acid decarboxylase 65-kilodalton isoform; HbA_1c_: glycated hemoglobin.

Figure 3 shows the individualized risk predictions for a patient at high risk for postdiagnosis DKA (Figure 3A) and low risk for postdiagnosis DKA (Figure 3B). For the patient in Figure 3A, the model shows the features contributing to high risk: worsening HbA_1c_ values from 9 to 21 months post diagnosis (9.8 → 10.1 → 10.6 →10.8), and high diabetes age of 8.39 years. For the patient in Figure 3B, the model identifies a low diabetes age of 1.44 years as the primary reason for low DKA risk. HbA_1c_ levels for this patient start at 6.7 at 9 months postdiagnosis and remain at 6.72 at the 12-month mark.

## Discussion

### Principal Findings

Approximately 18% of patients with T1D experience DKA after onset and yet there are few tools available to assist clinicians in assessing postdiagnosis DKA risk, either at the cohort level or at an individual level [39]. Our gradient-boosted ensemble of decision trees trained on a diverse cohort of 1787 patients with T1D has demonstrated the ability to predict DKA after onset with high accuracy, revealing insights into the features most predictive of high risk, and offering explainable risk scores at the level of an individual patient.

With an AUC of 0.80 (SD 0.04), a weighted *F*_1_-score of 0.78 (SD 0.04), and weighted precision and recall of 0.83 (SD 0.03) and 0.76 (SD 0.05) respectively, the model delivers performance similar to Food and Drug Administration–approved predictive computational tools for detecting cervical and breast cancer [40,41]. Using Shapley value analysis, the model identified diabetes age and HbA_1c_ at 12 months as the top 2 drivers of postdiagnosis DKA (Figure 1). Even more interesting, is the data-driven discovery of a “critical period” between 2.2 and 4.3 years of disease and an HbA_1c_ at 12 months between 8 and 10.3 that poses the greatest risk for postdiagnosis DKA, as revealed by the main-effects Shapley value plots. During this period, a sharp nonlinear rise in DKA risk (Figures 1B and 1C) suggests that the optimal window for preventive intervention may exist years prior to the adverse event. By clustering Shapley values using a hierarchical agglomerative clustering technique, we can cleanly stratify the population into 3 major risk classes: 30% in the low-risk group (5% risk of postdiagnosis DKA), 50% in the medium-risk group (20% risk of postdiagnosis DKA) and 20% in the high-risk group (48% risk of postdiagnosis DKA). Consistent with the high AUC scores, the model displayed clear separation between patients with T1D with no DKA postdiagnosis, and those with DKA postdiagnosis (P<.001; Figure 1A), holding promise for accurate identification of at-risk patients, with personalized risk scores highlighting individual patient-level factors that drive postdiagnosis DKA risk (Figure 3).

Our model, made interpretable by Shapley value analysis, provides insights into the key determiners of risk for postdiagnosis DKA, and elucidates the nonlinear relationships between key predictors and postdiagnosis DKA risk. Using the Shapley value framework, the model assesses risks at both the cohort and at the individual level, guiding the choice of therapeutic interventions.

### Comparison With Prior Work

Data-driven approaches to building predictive risk models are becoming important in clinical applications as prescriptive analytics and targeted personalized therapy become more readily available [28,42]. Recent models [22,23] for predicting patients at high risk for DKA have used logistic regression analyses to identify the top 3 features indicative of postdiagnosis DKA in pediatric T1D: most recent HbA_1c_, type of health insurance, and prior occurrence of DKA in the past 2 years. These models were qualitatively evaluated in a retrospective setting.

Our unique contribution is the design of an explainable predictive model for postdiagnosis DKA using one of the largest pediatric T1D cohorts studied in the literature. Our model’s predictive performance surpasses the state of the art on this problem (Williams et al [31]) on a similar patient cohort. It does so using variables collected on a patient with pediatric T1D during diagnosis, and routine clinic follow-ups for up to 24 months, and not measurements gathered from DKA hospitalization visits (which are fully correlated with our outcome variable). In addition, through our choice of model and statistical analysis using the Shapley value framework, we identify key risk factors predictive of postdiagnosis DKA at the population level and the individual level. We are able to reveal sharp changes in postdiagnosis DKA risk over time, identifying intervals for possible intervention. Finally, we perform risk stratification by automatically deriving risk clusters from Shapley values.

The ensemble model developed here has robust quantitative performance measures. It captures the heterogeneity inherent in the T1D population by building a set of weighted models, rather than a single linear model. Further, it can be operationalized as a predictive tool within existing EHR frameworks, allowing for better clinical management of pediatric T1D with enhanced resource allocation where specialized diabetes care is scarce [43,44].

Our model is derived from data spanning a decade in a large and diverse cohort of patients with pediatric T1D at a major tertiary-care children’s hospital. Data readily available in the EHR was included in the data input to the model. The model lets the data drive the selection of key predictors, thus eliminating human bias. The gradient-boosted ensemble method is key to predictive performance since (1) the relationships between predictors and the outcome variable are highly nonlinear (Figures 1B and 1C), precluding the use of simpler models such as logistic regression, and (2) there is significant variation among patients in the cohort, precluding the use of a one-size-fits-all model [32,33,45]. To our knowledge, this is the first deployment of such a model to predict DKA occurrence in a pediatric context. However, successful deployment of the model in a decision support context requires careful integration into clinical workflow.

### Limitations and Strengths

This study is a single-center, retrospective study with data limited to what was currently available in the EHR. We acknowledge that the EHR does not capture every patient characteristic that impacts clinical outcomes. Including data collected outside of the traditional health care environment, that is, remote patient monitoring data, and social determinants of health, can improve the predictive performance of our model.

We further acknowledge that DKA occurrences postdiagnosis are not always deterministically predictable, particularly in cases involving infection, illness, and instances of inadequate parental supervision.

In our study, the postdiagnosis DKA outcome in a patient with T1D is determined by hospitalization for DKA; this is a commonly used criterion in prior work on pediatric T1D [31]. However, it is possible that patients with mild cases of postdiagnosis DKA who did not require hospitalization, or were treated at a different facility are not accounted for in our outcome definition.

### Conclusions

We have built an explainable, predictive, machine learning model with potential for integration into clinical workflow. The model risk-stratifies patients with pediatric T1D and identifies patients at the highest postdiagnosis DKA risk using limited follow-up data starting from the time of diagnosis. The model identifies key time points and risk factors to direct clinical interventions at both the individual and cohort levels. The clinical import of our work is that the model can predict patients most at risk for postdiagnosis DKA and identify preventive interventions based on mitigation of individualized risk factors.

Future work includes further developing the model with data from multiple hospital systems, testing its generalizability across cohorts from other institutions, and prospectively studying whether it can assist clinicians target interventions to improve outcomes.

## Appendix

Multimedia Appendix 1 Supplementary figures and tables.

## Abbreviations

AUC: area under the receiver- operating characteristic curve
DKA: diabetic ketoacidosis
EHR: electronic health record
GAD65: glutamic acid decarboxylase 65-kilodalton isoform
HbA_1c_: glycated hemoglobin
ICA512: islet cell autoantigen 512
SHAP: Shapley additive explanations
T1D: type 1 diabetes
TCH: Texas Children’s Hospital
