# Supplementary material for: A Machine Learning Model for Risk Stratification of Postdiagnosis Diabetic Ketoacidosis Hospitalization in Pediatric Type 1 Diabetes: Retrospective Study
Source: JMIR Diabetes. 2024 Aug 7;9:e53338. doi: 10.2196/53338 (PMC11339561; doi:10.2196/53338)
Supplement: Multimedia Appendix 1 [file diabetes_v9i1e53338_app1.docx]

**Figure S1:** Protocol for five-fold stratified cross-validation model training and evaluation. The data set was divided into five equal-sized folds (1-5), with the ratio of postdiagnosis DKA and non-DKA patients being equal in all groups. Four of the folds were used for training a gradient-boosted ensemble, and the held-out fold (highlighted in yellow) was used for testing the ensemble. This procedure was repeated five times (Models A-E), each time with a different held-out fold, yielding five sets of performance measures (scores). The mean and standard deviation of these scores were used to characterize the ensemble model’s generalization performance.


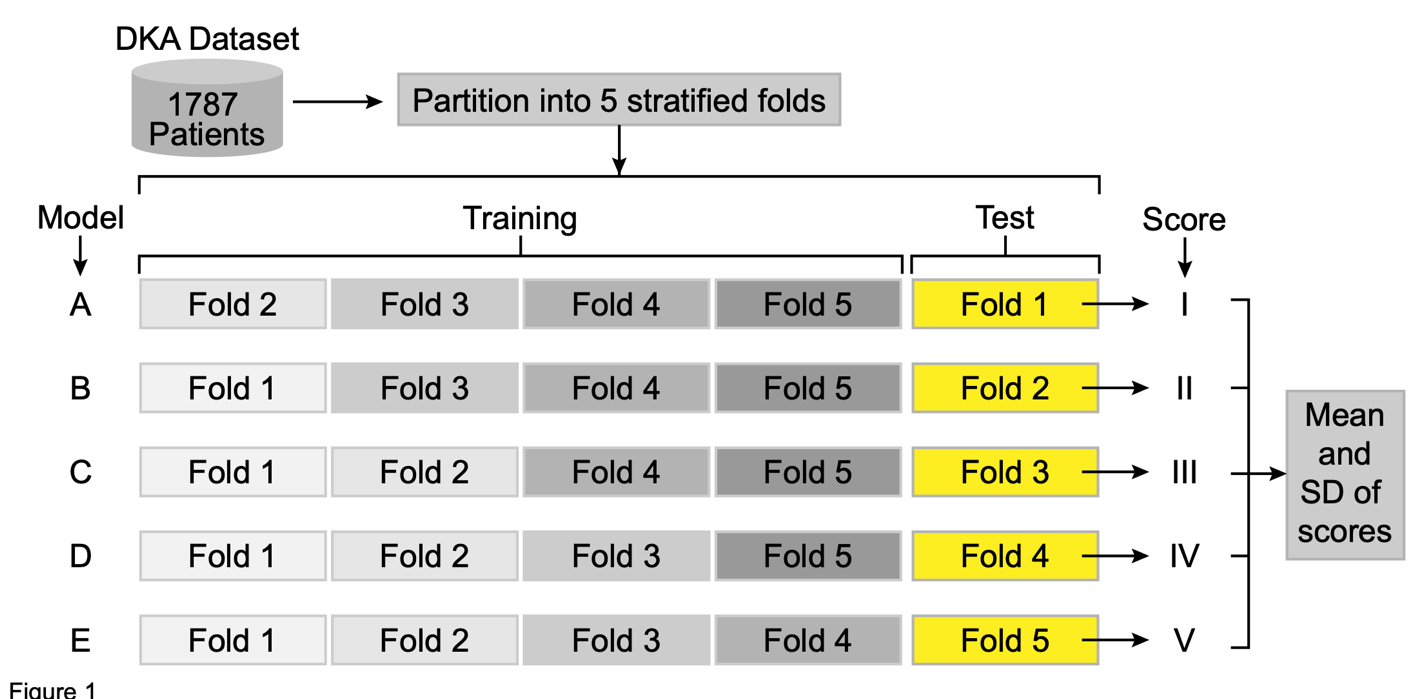


**Table S1**: Performance of the gradient-boosted model as a function of HbA1c values. The top row shows the model’s predictive power with all the demographic features, diabetes titers at diagnosis, C-peptide at diagnosis, DKA at onset, onset age, and diabetes age with a single baseline HbA1c measurement. The predictive power of the model increases with the addition of HbA1c at 3, 6, 9, 12, 15, and 18 months, with stabilization of model performance occurring with the inclusion of HbA1c values at 18 months and beyond.

| **HbA1c Values** | **AUC** | **F1** | **Precision** | **Recall** |
| --- | --- | --- | --- | --- |
| **baseline** | 0.75±0.05 | 0.69±0.07 | 0.81±0.02 | 0.66±0.10 |
| **3 months** | 0.76±0.06 | 0.70±0.08 | 0.81±0.03 | 0.67±0.10 |
| **6 months** | 0.77±0.06 | 0.72±0.08 | 0.81±0.03 | 0.69±0.10 |
| **9 months** | 0.78±0.06 | 0.72±0.07 | 0.81±0.02 | 0.69±0.09 |
| **12 months** | 0.79±0.05 | 0.76±0.06 | 0.82±0.02 | 0.73±0.08 |
| **15 months** | 0.79±0.05 | 0.76±0.05 | 0.82±0.02 | 0.74±0.07 |
| **18 months** | 0.80±0.04 | 0.78±0.05 | 0.83±0.02 | 0.76±0.07 |
| **21 months** | 0.80±0.04 | 0.78±0.04 | 0.83±0.02 | 0.76±0.07 |
| **24 months** | 0.80±0.04 | 0.78±0.04 | 0.83±0.02 | 0.76±0.07 |

**Table S2**: Sizes of the training, validation and test splits in the five-fold cross-validation training process with the cohort of 1787 patients of which 324 patients are positive – i.e., experience DKA postdiagnosis. Each entry is of the form Total (Negative examples/Positive examples)

| Fold | Training set | Validation set | Test set |
| --- | --- | --- | --- |
| 1 | 1286 (1053/233) | 143 (117/26) | 358 (293/65) |
| 2 | 1286 (1053/233) | 143 (117/26) | 358 (293/65) |
| 3 | 1287 (1054/233) | 143 (117/26) | 357 (292/65) |
| 4 | 1287 (1054/233) | 143 (117/26) | 357 (292/65) |
| 5 | 1287 (1054/233) | 143 (117/26) | 357 (292/65) |
